# Brief trauma therapy for occupational trauma-related PTSD/CPTSD in UK police

**DOI:** 10.1093/occmed/kqab075

**Published:** 2021-07-17

**Authors:** C Biggs, N Tehrani, J Billings

**Affiliations:** 1 Division of Psychiatry, University College London, London, UK; 2 Noreen Tehrani Associates, Twickenham, UK

**Keywords:** Complex PTSD, occupational trauma, police officers, PTSD, trauma therapy

## Abstract

**Background:**

Police are frequently exposed to occupational trauma, making them vulnerable to post-traumatic stress disorder (PTSD) and other mental health conditions. Through personal and occupational trauma police are also at risk of developing Complex PTSD (CPTSD), associated with prolonged and repetitive trauma. Police Occupational Health Services require effective interventions to treat officers experiencing mental health conditions, including CPTSD. However, there is a lack of guidance for the treatment of occupational trauma.

**Aims:**

To explore differences in demographics and trauma exposure between police with CPTSD and PTSD and compare the effectiveness of brief trauma-focused therapy between these diagnostic groups.

**Methods:**

Observational cohort study using clinical data from the Trauma Support Service, providing brief trauma-focused therapy for PTSD (cognitive behavioural therapy/eye movement desensitization and reprocessing) to UK police officers. Demographics, trauma exposure, baseline symptom severity and treatment effectiveness were compared between police with PTSD and CPTSD. Changes in PTSD, depression and anxiety symptoms were used to measure treatment effectiveness.

**Results:**

Brief trauma therapy reduced symptoms of PTSD, depression and anxiety. Treatment effectiveness did not differ between CPTSD and PTSD groups. Police with CPTSD exposed to both primary and secondary occupational trauma had poorer treatment outcomes than those exposed to a single occupational trauma type.

**Conclusions:**

Brief trauma-focused interventions are potentially effective in reducing symptoms of PTSD, depression and anxiety in police with CPTSD and PTSD. Further research is needed to establish whether additional CPTSD symptoms (affect dysregulation, self-perception and relational difficulties) are also reduced.

**Key learning points**

What is already known about this subject:

- Police are exposed to high levels of occupational trauma and have an increased risk of mental health difficulties including post-traumatic stress disorder (PTSD), depression and anxiety.
- National Institute for Health and Care Excellence recommends 8–12 sessions of trauma-focused cognitive behavioural therapy (TF-CBT) or eye movement desensitization and reprocessing (EMDR) for PTSD treatment. For complex PTSD (CPTSD), more sessions are suggested but there is no recommended number.

What this study adds:

- A significant number of police officers are experiencing CPTSD to occupational trauma.
- Brief 6- to 8-session TF-CBT or EMDR substantially decreases symptoms of PTSD, depression and anxiety in police with either PTSD or CPTSD.
- Police exposed to more than one occupational trauma type may respond less favourably to brief therapy.

What impact this may have on practice or policy:

- This highlights the need to consider CPTSD within policy and guidance for managing mental health difficulties within the police.
- This information will support occupational health professionals and psychologists in determining which police officers may benefit from brief therapy.

## Introduction

Police are at substantially increased risk of experiencing mental health difficulties, most notably post-traumatic stress disorder (PTSD) and depression [1]. This relates to unavoidable exposure to both direct (primary) and indirect (secondary) trauma in the profession [2]. Data released to the BBC through a freedom of information request showed a 16% increase in sick leave taken for mental health reasons in 2018–19 compared with 2014–15 [3].

Police services in the UK use Occupational Health Services to assess police fitness to work, provide advice and onward referral to support services as appropriate [4]. The level of support available nationally is however highly variable with no treatment guidelines on the treatment and management of occupational trauma [5].

The latest version of the WHO’s International Classification of Diseases (ICD-11) differentiates PTSD from complex post-traumatic stress disorder (CPTSD). CPTSD includes the three core symptoms of PTSD (re-experiencing, avoidance and perceived heightened current threat), as well as difficulties with affect regulation, self-perception (typical feelings of guilt or worthlessness) and relational difficulties. CPTSD is often associated with prolonged, repetitive or inescapable traumatic events [6].

National Institute for Health and Care Excellence (NICE) guidelines recommends 8–12 sessions of trauma-focused cognitive behavioural therapy (TF-CBT) or eye movement desensitization and reprocessing (EMDR) for PTSD treatment [7]. Both TF-CBT and EMDR help the person reprocess the trauma memory and include exposure as a core element.

There is not yet a NICE guideline for the treatment of CPTSD. The International Society for Traumatic Stress Studies (ISTSS) expert consensus survey showed strong clinical endorsement for a phase-based approach to CPTSD treatment [8]. This considers three treatment phases: engagement and stabilization; processing traumatic memories; and finally, functional reintegration, supporting the person to reclaim their life and develop skills for daily life. The duration of treatment is much longer than for PTSD [9].

Contradicting this, some studies have shown no difference in the effectiveness of trauma-focused interventions, when comparing people with PTSD with and without emotional regulation difficulties [10] and when comparing people with PTSD and CPTSD [11]. Critics therefore argue that the phase-based approach could needlessly delay access to evidence-based treatment for some individuals [12]. There is a need for further research into the treatment of CPTSD as the diagnostic category comes into formal use [13].

Given the nature, frequency and severity of trauma exposure in the police and potential impact of prior trauma exposure [14], police are arguably at significant risk of CPTSD as well as PTSD. A recent study showed the prevalence of CPTSD is greater than PTSD in UK police officers [15]. There is a financial imperative and statutory duty [16] to implement effective mental health treatment and prevention services for the police, not least given the importance of earlier intervention to achieve optimal outcomes for PTSD [17].

It is possible that both the nature of the trauma response [18] and appropriate treatment for occupational trauma may be different from non-occupational trauma. Current evidence suggests that longer treatment may be required for CPTSD [7], but this has not yet been investigated in CPTSD related to occupational trauma.

We used clinical data to evaluate the effectiveness of a service providing brief trauma-focused interventions to police officers with PTSD and CPTSD. A recent evaluation of the service programme shows benefits in emergency service workers with PTSD [19]; however, effectiveness for CPTSD has not yet been investigated.

Three core questions are addressed. Are there differences in demographics or trauma exposure between police with CPTSD compared with PTSD receiving treatment through the service? Are there differences in treatment effectiveness between police with CPTSD and PTSD? What is the influence of other variables including trauma exposure type on the effectiveness of the treatment programme overall, or specific to those with a CPTSD diagnosis?

## Methods

The Trauma Support Service offers a range of trauma interventions to help organizations including police services manage the impact of trauma on their employees. Access to the service is generally gained through Occupational Health Service referrals. The service offers 9–12 h of TF-CBT, EMDR or a combination of these therapies, delivered in 60- or 90-min sessions. Prior to referral, a battery of online health questionnaires is completed, including two measures for PTSD: Impact of Events Scale—Extended (IES-E) and International Trauma Questionnaire (ITQ). Police are referred to the service if one or both of these measures indicate a potential PTSD diagnosis. A clinical assessment is undertaken by a Health and Care Professions Council (HCPC) registered clinical or counselling psychologist. This confirms diagnosis and suitability for the service.

This study used clinical data from the Trauma Support Service to assess the effectiveness of the service for police with CPTSD when compared with police with PTSD. The following pre- and post-therapy measures were collected to measure changes in symptoms: IES-E measuring PTSD symptoms of re-experiencing, avoidance and arousal, a score greater than 50 indicating clinical diagnosis [20,21]; Goldberg Anxiety/Depression Scale (GADS) measuring symptoms of depression and anxiety, a score greater than 5 for either depression or anxiety indicating clinical diagnosis [22]; sense of coherence (SOC) measuring resilience through manageability, meaningfulness and comprehendability of adversity, validated across multiple cultures [23,24]. The 13-item version (SOC-13) was used in this study with the unidimensional score used in the analysis.

Additionally, an internally developed measure of perceived ability to manage trauma and work, using a five-point Likert scale (1 = very poor, 5 = excellent), to assess understanding of trauma symptoms; coping with the demands of the job; relationships with colleagues; relationship with manager; satisfaction with personal life; and ability to deal with own problems was used. Individuals were also asked to provide an estimate of their workplace functioning as a percentage of their full capacity before and after therapy and to complete a service satisfaction survey at the end of therapy.

Independent-sample *t*-tests and chi-squared tests were conducted to assess for differences in trauma exposure or demographics between the CPTSD and PTSD groups at baseline. Simple linear regression was used to assess the size of any differences in scores on measures completed at baseline.

Difference scores were calculated for each measure, from pre- and post-therapy scores. Simple linear regression analysis with each difference score as the outcome variable was used to assess differences in the effect of treatment on measures’ scores between the CPTSD and PTSD groups. Clinical cut-off scores for IES-E and GADS were used to identify and compare the proportion of clinical cases before and after treatment. Although all participants had a confirmed diagnosis of PTSD or CPTSD before treatment, some scored below the IES-E clinical cut-off. However, the measure provides an approximation for the change in the number of cases. In addition to diagnostic measures, a change in sense of coherence gives an indication of coping capacity and resilience to additional stressors. This is highly relevant in the context of police who are in an occupation with known psychological stressors and could give an indication of variation in coping between CPTSD and PTSD groups.

A supplementary regression analysis was conducted considering the CPTSD and PTSD groups separately, to investigate whether other variables including the type of therapy, age, gender or type of trauma exposure influenced treatment effectiveness. Occupational trauma was classified as primary trauma (direct exposure to a traumatic event), or secondary trauma (indirect trauma exposure such as reviewing crime materials or videos), or relationship trauma. Statistical power was sufficient for supplementary analysis; however, relationship trauma was excluded due to the small sample size, and occupational trauma re-categorized into primary trauma only, secondary trauma only or primary and secondary trauma exposure.

The acceptability of the service was assessed through the service satisfaction survey, with responses compared between CPTSD and PTSD groups.

As a service evaluation, additional ethical approval was not required for this study. Analysis was conducted using IBM SPSS Statistics 25. Figures were produced using Prism 8, by GraphPad.

## Results

The sample consisted of 162 police personnel; 51% male, 49% female. Age ranged from 23 to 65 (mean 42 years old). The mean amount of treatment provided was 10 h. The intervention chosen by the client and therapist was TF-CBT (44%), EMDR (40%), combined approach (16%). The majority (78%) had been exposed to more than one type of trauma, and 61% had been exposed to trauma in more than one area of life (childhood, adult non-occupational trauma and occupational trauma). Ninety (56%) met the criteria for CPTSD and 72 (44%) for PTSD, diagnosed through clinical interview and ITQ [25]. About 95% also met the criteria for an anxiety disorder and 88% for depression, according to GADS [22].

**Figure 1.**
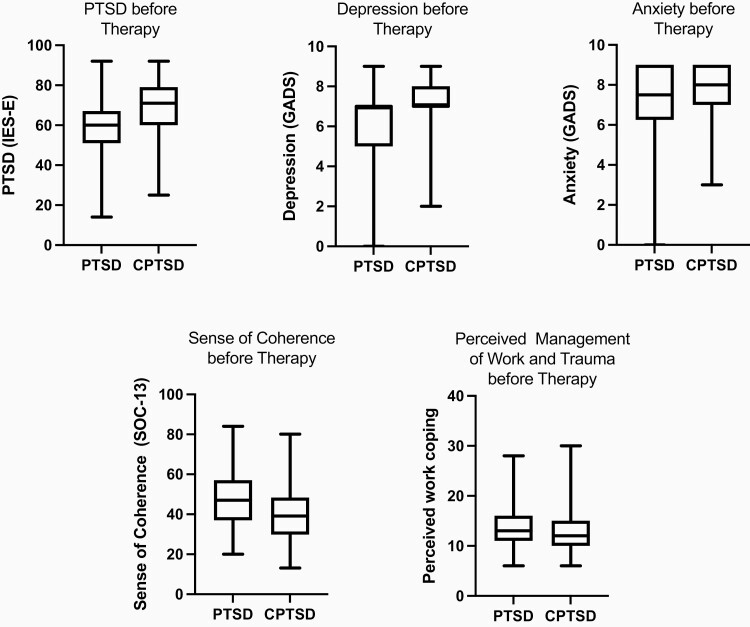
Boxplots showing the distribution of scores at baseline compared between PTSD and CPTSD groups for PTSD, depression, anxiety, sense of coherence and perceived management of trauma and work.

Comparing police personnel presenting with CPTSD to those with PTSD, there were no demographic differences. The mean number of separate types of trauma exposure disclosed was slightly greater in those with CPTSD compared with PTSD (2.63:2.31), as was the proportion exposed to childhood or adult non-occupational trauma. However, these differences were not statistically significant (Table 1). Of the 36 police persons who disclosed trauma in childhood, adult non-occupational and occupational trauma, two-thirds met the criteria for CPTSD. Conversely, 23 police with CPTSD disclosed only occupational trauma. Simple linear regression showed very strong evidence (*P* < 0.001) that the severity of PTSD, depression and anxiety symptoms was all greater in the CPTSD group compared with the PTSD group at baseline. There was also evidence (*P* < 0.05) that sense of coherence was lower at baseline in the CPTSD group (Table 2 and Figure 1).

**Table 1.** Comparison of demographic variables and the number and proportion of police who declared each type of trauma exposure between CPTSD and PTSD groups

	CPTSD, *n* (%)	PTSD, *n* (%)	χ^2^ (df)	*P* value
Childhood trauma				
Child trauma	32 (37)	20 (28)	1.45 (1)	NS
Child abuse	20 (23)	15 (21)	0.11 (1)	NS
Child loss	4 (5)	3 (4)	0.02 (1)	NS
Any childhood trauma	46 (53)	31 (43)	1.52 (1)	NS
Adulthood trauma				
Adult trauma	34 (39)	21 (29)	1.71 (1)	NS
Adult relationships	22 (25)	15 (21)	0.44 (1)	NS
Adult abuse	1 (1)	3 (4)	1.46 (1)	NS
Any adult trauma	49 (56)	33 (46)	1.74 (1)	NS
Occupational trauma				
Primary trauma	59 (68)	44 (61)	0.78 (1)	NS
Secondary trauma	43 (49)	36 (50)	0.01 (1)	NS
Work relationships	14 (16)	9 (13)	0.41 (1)	NS
>1 Occupational trauma type	34 (44)	25 (40)	0.28 (1)	NS
Demographic variables				
Gender (proportion male)	49 (54)	41 (57)	2.08 (1)	NS
Age: Mean (SD)	42.3 (8.03)	41.7 (8.77)	–	NS

Categorical variables were assessed with chi-squared testing. Continuous variables were assessed using an independent-sample *t*-test.

**Table 2.** Results from simple linear regression analyses conducted to assess differences in baseline scores on each of the following measures: PTSD (measured by IES-E), depression (measured by GADS), anxiety (measured by GADS), sense of coherence, and perceived management of trauma and work (measured by internally developed scale)

	Score at baseline				
	CPTSD, mean (SD)	PTSD, mean (SD)	Difference in means	95% confidence interval	*P* value
PTSD (IES-E)	69.81 (12.47)	58.65 (14.97)	11.16	6.90 to 15.42	<0.001
Depression (GADS)	7.20 (1.33)	6.07 (1.96)	1.13	0.62 to 1.64	<0.001
Anxiety (GADS)	7.91 (1.34)	7.31 (1.76)	0.61	0.13 to 1.09	<0.05
Sense of coherence	41.28 (14.08)	47.93 (14.77)	−6.65	−11.15 to −2.16	<0.05
Perceived management	12.91 (4.25)	13.49 (3.95)	−0.58	−1.89 to 0.72	NS

In each case, the baseline score was the outcome variable and diagnosis (CPTSD or PTSD) was the exposure variable.

In the full sample, two-tailed paired *t*-tests showed strong evidence of treatment effectiveness in reducing symptoms of PTSD (baseline M (SD): 63.62 (15.51); follow-up M (SD): 33.49 (21.18); *P* < 0.001), depression (baseline M (SD): 6.5 (1.95); follow-up M (SD): 3.3 (2.5), *P* < 0.001), and anxiety (baseline M (SD): 7.4 (1.8); follow-up M (SD): 4.4 (2.7.); *P* < 0.001).

There was no difference in the decrease in symptoms of PTSD, depression or anxiety when comparing the CPTSD and PTSD groups, according to simple linear regression analysis (Table 3).

**Table 3.** Difference in treatment effectiveness between CTPSD and PTSD groups assessed through simple linear regression analysis with the change of symptoms score as outcome variable and PTSD/CPTSD diagnostic group as exposure variable

	Change in score				
Variable	CPTSD, mean (SD)	PTSD, mean (SD)	Difference in means	95% confidence interval	*P* value
PTSD (IES-E)	34.11 (21.80)	28.93 (22.99)	5.18	−1.85 to 12.21	NS
Depression (GADS)	3.69 (2.75)	3.01 (2.95)	0.68	−0.22 to 1.57	NS
Anxiety (GADS)	3.43 (3.07)	3.07 (2.93)	0.36	−0.59 to 1.31	NS
Sense of coherence	13.96 (19.68)	8.99 (16.63)	4.97	−0.83 to 10.77	NS
Perceived management	9.84 (4.74)	9.79 (4.79)	0.05	−1.52 to 1.62	NS

**Figure 2.**
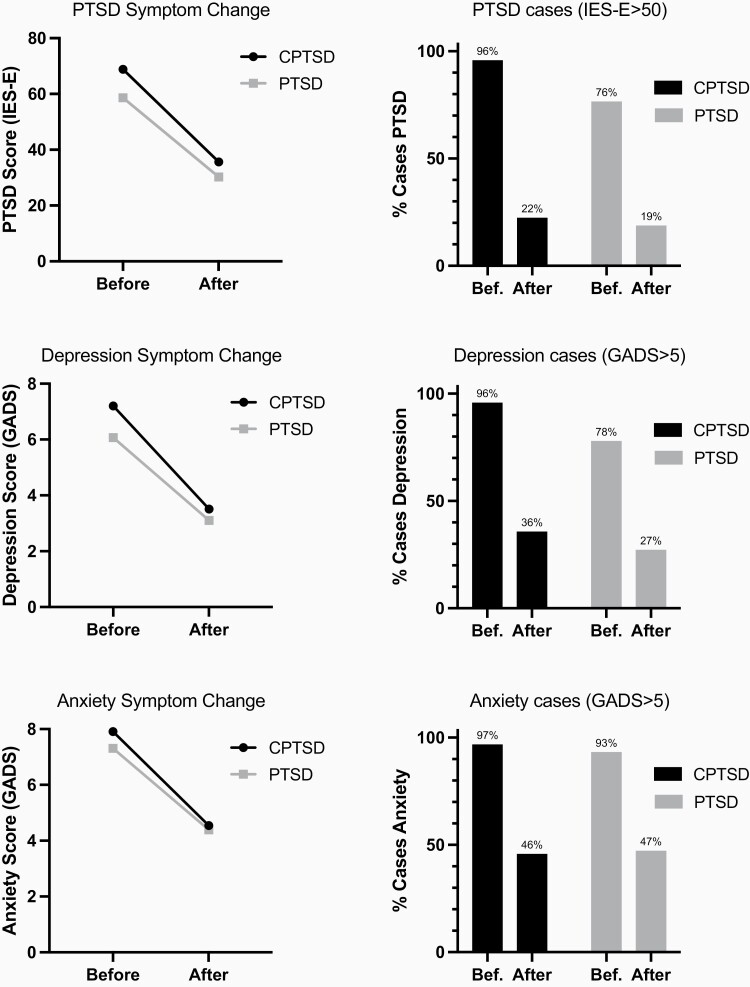
Left: Change in mean score before and after therapy for PTSD (measured by IES-E), anxiety (measured by GADS) and depression (measured by GADS), with a comparison between the CPTSD and PTSD group. Right: Change in proportion of cases before and after therapy for PTSD, anxiety and depression, separated by CPTSD or PTSD group. Case criteria: PTSD (IES-E >50), anxiety (GADS anxiety >5), depression (GADS depression >5). IES-E clinical cut-off is used as an indication of treatment effect but not a diagnostic tool. All met the criteria for PTSD or CPTSD diagnosis at baseline. Those below IES-E criteria at baseline are false-negative results.

The difference in the proportion of cases moving below diagnostic criteria between PTSD and CPTSD groups, as approximated by IES-E and GADS clinical cut-off scores, was compared to assess clinical significance. About 77% of those meeting case criteria for PTSD at baseline, no longer met criteria after treatment, and there was no difference between CPTSD and PTSD groups. For both depression and anxiety, over 50% of cases at baseline no longer met the criteria after treatment. A slightly larger proportion of the CPTSD group remained above diagnostic criteria for depression after treatment (36%), compared with the PTSD group (27%), corresponding with a greater proportion of cases at baseline (Figure 2).

A secondary linear regression analysis was conducted considering the influence of other variables on the treatment effect. Within the CPTSD group improvements in PTSD, depression and anxiety symptoms were smaller for those who experience both primary and secondary occupational trauma than a single form of occupational trauma. There was also strong evidence that those who received a combined treatment had smaller improvements in symptoms of depression and anxiety than TF-CBT or EMDR. There was week evidence that older age was associated with smaller decreases in PTSD symptoms, although this was eliminated by controlling for baseline symptoms severity. Other variables including prior exposure to childhood or adult non-occupational trauma did not affect treatment outcomes (Table 4). This analysis was repeated in the PTSD group where there was no evidence of a difference in treatment effectiveness associated with any of the variables.

**Table 4.** Difference in treatment effect associated with therapy type, occupational trauma, gender and age for police personnel with a CPTSD diagnosis

Variable	Mean (SD)	Effect size	95% confidence interval	*P* value
PTSD symptom change (IES-E)				
*Therapy type*				
TF-CBT	33.75 (23.71)	–	–	–
EMDR	35.00 (19.79)	1.25	−8.74 to 11.24	NS
Combined	32.69 (22.76)	−1.06	−15.05 to 12.93	NS
*Occupational trauma*				
Primary trauma	39.17 (20.16)	–	–	–
Secondary trauma	42.08 (22.96)	2.91	−11.64 to 17.44	NS
Primary and secondary	26.13 (22.89)	−13.04	−24.38 to −1.70	<0.05
*Gender*				
Male	34.31 (20.93)	–	–	–
Female	33.88 (23.06)	0.43	−8.79 to 9.65	NS
*Age*	34.11 (21.80)	−0.65	−1.21 to −0.09	<0.05
Depression symptom change (GADS)				
*Therapy type*				
TF-CBT	4.00 (2.49)	–	–	–
EMDR	4.14 (2.45)	0.14	−1.05 to 1.32	NS
Combined	1.46 (3.41)	−2.54	−4.20 to −0.88	<0.01
*Occupational trauma*				
Primary trauma	4.55 (2.16)	–	–	–
Secondary trauma	4.38 (2.16)	−0.17	−1.91 to 1.57	NS
Primary and secondary	2.67 (2.89)	−1.89	−3.24 to −0.53	<0.01
*Gender*				
Male	3.53 (2.85)	–	–	–
Female	3.88 (3.06)	−0.35	−1.51 to 0.82	NS
*Age*	3.69 (2.75)	−0.07	−0.14 to 0.00	NS
Anxiety symptom change (GADS)				
*Therapy type*				
TF-CBT	3.73 (2.60)	–	–	–
EMDR	3.84 (3.00)	0.11	−1.24 to 1.46	NS
Combined	1.38 (3.93)	−2.34	−4.23 to −0.45	<0.05
*Occupational trauma*				
Primary trauma	4.07 (2.34)	–	–	–
Secondary trauma	4.31 (2.87)	0.24	−1.65 to 2.13	NS
Primary and secondary	2.40 (3.22)	−1.67	−3.14 to −0.20	<0.05
*Gender*				
Male	3.69 (2.95)	–	–	–
Female	3.12 (3.20)	0.57	−0.72 to 1.86	NS
*Age*	3.43 (3.07)	−0.04	−0.12 to 0.04	NS

Sense of coherence, perceived ability to manage trauma and work and service satisfaction were also compared. In the full cohort two-tailed *t*-tests showed strong evidence of an increase in sense of coherence (baseline M (SD): 44.21 (14.77); follow-up M (SD): 55.99 (14.28), *P* < 0.001) and an increase in the perceived ability to manage trauma and work (baseline M (SD): 13.36 (4.10); follow-up M (SD): 23.17 (4.01), *P* < 0.001). Police were also asked to estimate their functioning at work as a percentage of their full capacity. Two-tailed paired *t*-tests showed strong evidence (*P* < 0.001) of an increase in perceived functioning from 34% to 70% across the full cohort. There was no evidence of a difference between CPTSD and PTSD groups for any of these measures. Satisfaction with the service, therapist and delivery was extremely high (average 95% total satisfaction), with no difference between diagnostic groups.

## Discussion

This study shows that a brief trauma-focused intervention can be effective in treating PTSD, depression and anxiety for police personnel with both CPTSD and PTSD diagnoses. The baseline severity of symptoms of PTSD, depression and anxiety was greater in police with CPTSD than PTSD. Despite this, there was no evidence of a difference in treatment effectiveness between those with CPTSD and PTSD and the percentage with symptoms above the diagnostic threshold after therapy was comparable. The majority of police officers had had exposure to more than one type of trauma; however, within both the CPTSD and PTSD group there were police who only disclosed occupational trauma. Police with CPTSD who reported exposure to primary and secondary occupational trauma had smaller symptom improvements, as did those receiving the combined treatment approach. Service satisfaction and perceived ability to manage trauma and work were favourable showing the service was acceptable and practically useful to police.

There are strengths to this study. As the study uses clinical service data there is no response bias and data was available for all who received the service. There is also very little missing data and reliable and validated measures were used. It is one of the first studies directly comparing treatment effectiveness between PTSD and CPTSD and is evaluating a brief and financially viable service. The findings also have immediate clinical value and utility to the service provider and police occupational health services on the effectiveness of their current provision.

There are also limitations. The service is provided to police personnel in high-risk roles or deemed to be in need of support by managers; therefore, this sample cannot be assumed to be representative of all police officers. Access to the service is also based on clinical assessment of suitability. This could inflate the apparent effectiveness of the service and limits the generalizability of the findings. It is possible that those with the most severe or sub-threshold symptoms were not included, so the sample may not be representative of the wider police populations’ mental health needs. All measures are self-report, completed online prior to the service and then in or immediately after the final therapy session. This brings limitations, highlighted by the discrepancies between ITQ and IES-E measures in determining PTSD cases. CPTSD-specific symptoms were not assessed after treatment. There are also a number of therapists delivering the interventions, and it was not possible to control for variation between therapists.

The main implication of this study is the potential effectiveness of a brief trauma-focused intervention for PTSD and CPTSD in police personnel. This contradicts the ISTSS recommendation for a longer phase-based treatment approach for CPTSD [8] and is consistent with recent findings from the Psychotrauma Expertise Centre comparing treatment outcomes for patients with CPTSD to PTSD [11]. While it may not be generalizable to all police with CPTSD, the service provides a financially viable, time-efficient option for supporting some police with CPTSD. Increasing access to brief interventions in coordination with existing health services and employers has also been utilized in a recent programme for the US Military where it appears to be improving access to timely treatment and identification of PTSD symptoms [26,27].

Contrary to our expectations, based on the association between childhood trauma and CPTSD in the literature [28], there was no association between exposure to non-occupational adult or childhood trauma and a CPTSD diagnosis or poorer treatment outcomes. About 26% of the police with CPTSD only reported exposure to occupational trauma. Although this may be subject to recall bias, it is consistent with previous latent profile analyses of CPTSD and PTSD where 20% experiencing a single traumatic event develop CPTSD and 23% with a history of child abuse, PTSD [29].

There were no differences in treatment response for police exposed to primary or secondary trauma in isolation. The small improvement in symptoms for police with CPTSD exposed to both primary and secondary occupational trauma may therefore be an indication of the cumulative impact of occupational trauma. This would also explain the small effect of older age on treatment response. A large-scale survey of UK Police officers, also using the ITQ, found a higher prevalence of CPTSD than PTSD and that this was associated with a greater total number of years of trauma exposure, as well as the nature of trauma and rank of the officer [15]. It appears possible that the introduction of the ITQ to assess for CPTSD has enabled the identification of police with CPTSD symptoms who would not previously have been identified. CPTSD or PTSD in response to occupational trauma may be different from non-occupational trauma.

Finally, those who received a combined (TF-CBT and EMDR) treatment had a smaller change in symptoms. This may be the result of therapeutic drift, where therapists move away from the treatment protocol and fail to deliver core elements of the evidence-based intervention, decreasing treatment efficacy [30]. However, there may be confounding factors influencing this choice of treatment, such that combined treatment is offered to the most complex cases who are not benefitting as much from standard treatment.

There is a need for further research to determine whether this brief trauma-focused therapy is effectively treating CPTSD-specific symptoms, and whether symptom-level improvements are maintained in the longer term. Further research into the potential cumulative effect of occupational trauma is needed to understand police personnel’s varying treatment and support needs throughout their careers. Alongside this, it would be beneficial to consider the current role and rank of the officer and how this may affect trauma response and treatment effectiveness. Further feasibility studies are needed to ascertain the most effective treatment for occupational trauma and how PTSD and CPTSD treatment in response to occupational trauma may differ from non-occupational trauma.

This study highlights the needs of police personnel, showing that their exposure to occupational trauma can lead to CPTSD as well as PTSD, depression and anxiety. However, in this study both police with CPTSD and PTSD responded well to a brief trauma-focused intervention, providing an affordable option for police services to provide timely support to their employees.
